# Seroprevalence of *Brucella abortus* and *Leptospira hardjo* in cattle

**DOI:** 10.14202/vetworld.2015.217-220

**Published:** 2015-02-23

**Authors:** S. Jegaveera Pandian, Pradeep Kumar Ray, P. C. Chandran, Manoj Kumar

**Affiliations:** 1 Division of Livestock and Fisheries Management, ICAR Research Complex for Eastern Region, Patna, Bihar, India; 2 Department of Microbiology, Bihar Veterinary College, Patna, Bihar, India

**Keywords:** brucellosis, cattle, infertility, leptospirosis, seroprevalence

## Abstract

**Aim:**

The aim was to assess the seroprevalence of *B. abortus* and *Leptospira hardjo* in the cattle population of Bihar, this work was carried out.

**Materials and Methods:**

Randomly selected 450 cattle from nine districts of Bihar were serologically screened for antibodies against *L. hardjo* and *B. abortus*. DAS-ELISA for leptospira and AB-ELISA for brucella were carried out. Based on the results prevalence in each district and the state are reported herewith.

**Results and Discussion:**

In this study, it was found that the seroprevalence of *L. hardjo* was 9.11% and that of *B. abortus* was 12.2% in Bihar. Indigenous cattle were found to be less susceptible to leptospirosis and brucellosis even though they accounted for 83.11% of the study population.

**Conclusion:**

Although there was no acute disease, antibodies detected against *L. hardjo* and *B. abortus* in the cattle population indicated the presence of chronic and subclinical infection, which could challenge the fertility of the animals.

## Introduction

Brucellosis is a potent zoonotic disease with global presence [1]. In cattle, brucellosis is mainly caused by *B. abortus*, characterized by abortion, still births, retained placenta, infertility and economic loss [2]. There are many species of brucella that affect both animal and human beings. The prevalence of brucellosis in animals and human beings is being reported in the literature frequently [3,4].

Leptospirosis is an occupational zoonotic disease reported from all over the world. It is caused by serovars of *Leptospira interrogans* in animals and human beings. *L. hardjo* is most commonly affecting cattle. Being a host-adapted serovar, it does not cause acute disease in cattle. However, because of more susceptibility and chronic course of the disease, infection with *L. hardjo* results in reproductive problems in cattle [5].

Many countries have recommended and enacted periodical screening for brucellosis in farm animals. From Bihar, there was no recent study on the prevalence of these diseases. Further, brucellosis and leptospirosis require surveillance to contain them and adapt policy decision in public health aspects. In order to assess and update the status of seroprevalence of *B. abortus* and *L. hardjo*, this work was undertaken.

## Materials and Methods

This study involved randomly selected cattle reared in nine districts of Bihar. Randomization was adhered in the selection of districts, unorganized farms, and animals. The information regarding the age, breed, sex, vaccination status and physiological status were collected. A total of 450 cows were studied during the period 2008-2010. Serum samples were collected as per standard procedure. From Patna district, samples were collected from two organized crossbred cattle farms. Serum samples were stored at −20°C till assay procedure.

### Approval of Animal Ethics Committee

As per CPCSEA guidelines, study involving clinical samples does not require approval of Institute Animal Ethics Committee.

### ELISA for B. abortus

Bovine brucellosis Avidin-Biotin ELISA kit was procured from Project Directorate on Animal Disease Monitoring and Surveillance (PDADMAS), Bengaluru and the ELISA protocol was followed precisely as per the instructions of kit developer. The absorbance of wells was read at 492 nm using a Microscan^®^ ELISA microplate reader (ECIL, Lucknow, India) and the percent positivity (PP) value was calculated as below. Based on the recommended cut-off PP value (40%), results were interpreted.


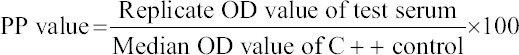


### ELISA for L. hardjo

Double Antibody Sandwich-ELISA (DAS-ELISA) kit was procured from Linnodee, Northern Ireland and the procedure in the pack insert was precisely followed. The Linnodee Bovine Leptospira ELISA kit can detect the antibody response to a lipopolysaccharide outer envelope epitope common to both *Leptospira borg petersenii* serovar *hardjo* (Type *Hardjo bovis*) and *Leptospira interrogans* serovar *hardjo* (Type *Hardjo prajitno*). The optical density (OD) of wells was measured at 450 nm using a Microscan^®^ ELISA microplate reader (Electronics Corporation of India Limited [ECIL], Lucknow, India). The sample value related to positive control value (S/P ratio) was calculated using the formula given below.


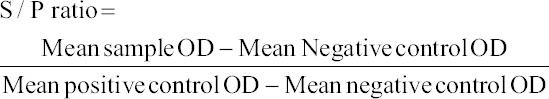


Samples with an S/P ratio of > 0.12 were considered positive and < 0.05 were considered negative. S/P ratio between 0.05 and 0.12 were considered inconclusive and retested.

## Results

The cattle population comprised of Jersey crossbred (9.33%), indigenous (83.11%) and Holstein cross (7.56%). The mean age of the cattle population was 4 years. Based on the information collected, it was found that 20.89% were pregnant, 75.11% were non-pregnant cows and 4% were heifers. None of the animal was vaccinated for leptospirosis and brucellosis. It was observed that both leptospirosis and brucellosis were more prevalent in Jersey crossbred animals. No sample was tested positive for both the diseases together in ELISA.

The observed district-wise prevalence of *L. hardjo* and *B. abortus* is presented in Table-1. The overall prevalence rate among cattle in Bihar was 9.11% for leptospirosis and 12.2% for brucellosis. The observed ranges of seroprevalence of leptospira and brucella antibodies were 0 - 34.92% and 5.38 - 25.4%, respectively. The breed-wise prevalence is given in Table-2.

**Table-1:** District-wise seroprevalence of *L. hardjo* and *B. abortus* in Bihar.

Districts	Number of samples	*L. hardjo* Number of positives (%)	*B. abortus* Number of positives (%)
Arwal	31	2 (6.45)	4 (12.90)
Patna	63	22 (34.92)	16 (25.4)
Katihar	93	3 (3.23)	5 (5.38)
Purnia	33	7 (21.21)	7 (21.21)
Darbhanga	68	5 (7.35)	5 (7.35)
Begusarai	73	0	5 (6.85)
Jamui	41	1 (2.44)	4 (9.76)
Khagaria	13	0	1 (7.69)
Saharsa	35	1 (2.86)	8 (24.24)
Bihar	450	41 (9.11)	55 (12.2)

L. hardjo=Leptospira hardjo, B. abortus=Brucella abortus

**Table-2:** Breed-wise incidence of *Leptospira* and *Brucella* in cattle.

Breeds	*Leptopspira hardjo* (%)	*Brucella abortus* (%)
Jersey cross	40.00	36.59
Holstein cross	34.55	29.27
Indigenous	25.45	34.15

## Discussion

Brucellosis remains to be very common in extensive and transhumance system of management [6,7]. It is an established fact that cattle aged beyond 3 years are more susceptible to brucellosis than young animals [6,8,9]. The mean age of cows in this study was 4 years. Hence, the seroprevalence of brucellosis is more in these animals. Comparing to high lands, animals maintained in low lands are very vulnerable to brucellosis. Lesser prevalence of brucellosis was reported from Bangladesh (2.66%), Pakistan (3.68%) and Eriteria (2.77%) and other developing countries [3,10-12]. Many authors predicted an alarming situation on the emergence of brucellosis. Geographical area, herd size, breed and contact/proximity to wild life also play a vital role in the seropositivity for brucellosis in cattle [13]. As, the present study area is located in the basin of Ganges and its tributaries, higher prevalence recorded could be attributed to low- lying plain land morphology.

In a serological screening for brucellosis in 12 Indian states using AB-ELISA, it was observed 8.8% cattle were found positive [14]. Crossbred cattle were found to be more seropositive for brucellosis than exotic and local breeds [15]. Whereas, seroprevalence of brucellosis in Punjab and Kerala was 12.09% and 4.02% [16,17] respectively. Significantly higher seroprevalence of brucella and leptospira were observed in two organized cattle farms in Patna district. In agreement with this finding, a higher prevalence of brucellosis was observed in organized farms than in cattle owned by individual farmers [18]. In 1990, seroprevalence of bovine brucellosis in Bihar was 19% when assessed using standard tube agglutination test [19]. Although there was no phenomenal increase in the seroprevalence, positive reactors in the unvaccinated population indicate infected and carrier animals in the population.

Seroprevalence of leptospirosis was associated with age but not with breed and health status [20]. In this study, the highest prevalence of *L. hardjo* was observed in Jersey crossbred cows although they were only 9.33% of the total study population. The composition of the population could very well alter the breed predisposition for any disease. This might also be reasoned out that indigenous cattle generally remain resistant and unsusceptible to common infections. Highest seroprevalence of leptospirosis was observed in 3-5 years of age [20]. Similarly, as the mean age was 4 years in this study, higher prevalence of leptospiral antibodies was observed.

Seroprevalence of *L. hardjo* was 3.50% and 1.19% respectively in Nigeria and Iran [21,22]. Looking at the global scenario, very low prevalence of leptospirosis was reported from various parts of the world. However in India, many states register higher prevalence of leptopspira. In cattle suspected for leptospirosis, 67.15% seropositivity was observed in the southern state of erstwhile Andhra Pradesh [23]. In Odisha, it was 42.5% [20]. Comparing to southern states, a lesser prevalence was observed in Bihar as the environmental determinants like soil pH might not be conducive for the survival of leptospires [5]. In this study, a total prevalence rate of 9.11% in Bihar and a highest herd prevalence rate of 34.92% was observed in Patna district. This might due to sampling from organized farms, which comprised only crossbred animals. Similarly, a positive correlation between the mean herd size and mean herd antibody levels was reported [24]. Higher prevalence in organized crossbred cattle farms could be attributed to the fact that infected animals in a confined population increase the risk of spread. However, in human beings, *L. hardjo* is the most common cause of acute febrile illness because of host non-adaptation [5]. Hence, it is advisable to zero in on the points of spread and control of leptospirosis.

## Conclusions

In the present study to assess the seroprevalence of *L. hardjo* and *B. abortus* in cattle, it was found that 9.11% of cattle maintained in Bihar carried antibodies for leptospirosis, and 12.2% had antibodies for brucellosis. Although there was no acute disease, antibodies detected against *L. hardjo* and *B. abortus* in the cattle population indicated the presence of chronic and subclinical infection, which could challenge the fertility of the animals.

## Authors’ Contributions

SJP: Principal investigator of the project, planning, sample collection, assay and manuscript preparation, PKR, PCC, MK: Co-investigators involved in sample collection and assay. All authors read and approved the final manuscript.
